# A systematic review and physiology of pulmonary artery pulsatility index in left ventricular assist device therapy

**DOI:** 10.1093/icvts/ivad068

**Published:** 2023-05-12

**Authors:** Ivan H W Yim, Ayisha M Khan-Kheil, Nigel E Drury, Hoong Sern Lim

**Affiliations:** Department of Cardiac Surgery, Queen Elizabeth Hospital Birmingham, Birmingham, UK; Department of Cardiology, Queen Elizabeth Hospital Birmingham, Birmingham, UK; Department of Cardiac Surgery, Queen Elizabeth Hospital Birmingham, Birmingham, UK; Department of Cardiology, Queen Elizabeth Hospital Birmingham, Birmingham, UK; Institute of Cardiovascular Sciences, University of Birmingham, Edgbaston B15 2TT, UK

**Keywords:** Left ventricular assist device, Heart failure, Right heart failure, Pulmonary artery pulsatility index

## Abstract

**OBJECTIVES:**

Right heart failure (RHF) is a major complication following left ventricular assist device (LVAD) implantation. Pulmonary artery pulsatility index (PAPi) has been evaluated as a haemodynamic marker for RHF, but PAPi is dependent on pulmonary vascular resistance (PVR). We conducted a systematic review to assess the relationship between PAPi and RHF and death in patients undergoing LVAD implantation and examined the relationship between PAPi cut-off and PVR.

**METHODS:**

We searched PubMed, EMBASE, CENTRAL and manually screened retrieved references to identify all clinical studies reporting PAPi in adult patients with a durable LVAD. Eligibility criteria were prespecified and 2 reviewers independently screened and extracted data; the Newcastle–Ottawa Scale was used to assess quality of non-randomized studies. This study was prospectively registered on PROSPERO (CRD42021259009).

**RESULTS:**

From 283 unique records, we identified 16 studies reporting haemodynamic assessment in 20 634 adult patients with an implanted durable LVAD. Only 2 studies reported on mortality and in both, a lower PAPi was significantly associated with death. Fifteen studies reported RHF data and, in 10 studies, a lower PAPi was significantly associated with RHF. Six studies reported on PAPi cut-offs ranging from 0.88 to 3.3; and the cut-offs were directly related to PVR (*r* = 0.6613, *P* = 0.019).

**CONCLUSIONS:**

Lower PAPi was associated with RHF and death following LVAD implantation, but a single PAPi cut-off cannot be defined, as it is dependent on PVR.

**Figure:**
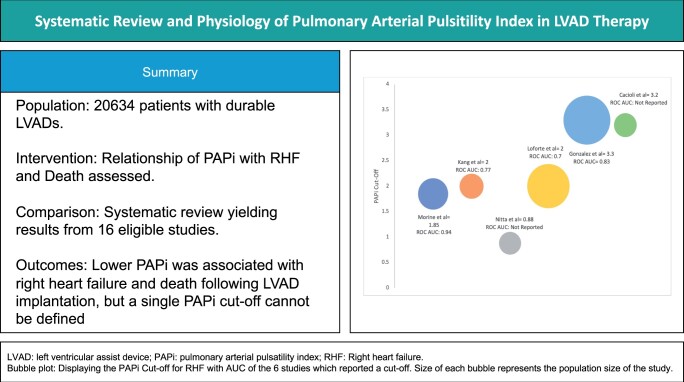

## INTRODUCTION

Durable left ventricular assist devices (LVAD) have become an established therapy in patients with end-stage heart failure. Outcomes of LVAD therapy have improved with the introduction of the magnetically levitated centrifugal continuous-flow device [1], but early right heart failure (RHF) remains a major cause of morbidity and mortality following LVAD implant [2]. Therefore, preoperative assessment of the risk of RHF is central to the selection of patients for LVAD therapy.

A number of haemodynamic parameters derived from pulmonary artery catheterization have been used to assess the risk of RHF. Of these, pulmonary artery pulsatility index (PAPi) has been shown to be an independent predictor of mortality due to RHF in acute myocardial infarction, pulmonary arterial hypertension, heart failure, heart transplantation and cardiogenic shock [3–7]. In general, lower PAPi is associated with higher the risk of RHF. PAPi is defined as the ratio of pulmonary artery pulse pressure (PP) to right atrial (or central venous) pressure. Pulmonary artery PP is a function of stroke volume (SV) and pulmonary artery compliance (PAC), and the latter has a hyperbolic relationship with pulmonary vascular resistance (PVR) [8]. On this basis, we hypothesized that the PAPI cut-off associated with RHF would be dependent on the PVR (increase with PVR).

This systematic review first assessed the relationship between PAPi, RHF and death following LVAD implantation. Second, we evaluated the relationship between the reported PAPi cut-off and PVR from the studies identified in this systematic review.

## METHODS

### Ethics statement

As this is a systematic review, ethics committee review was not applicable. However, this study was formally reviewed and was prospectively registered on PROSPERO (CRD42021259009). The search results are reported in accordance with the PRISMA statement [9]. All eligibility criteria and search strategies were prespecified.

### Eligibility

All studies reporting measurement of PAPi in adult patients with a durable LVAD, defined as an intra- or extracorporeal device implanted in the left ventricle for the treatment of advanced heart failure, irrespective of treatment intention (destination therapy or bridge to or transplantation), and published in the English literature were included. Studies were excluded if PAPi was not reported, or data on mortality or RHF were not reported. Studies reported only as a conference abstract were excluded due to insufficient data for analysis.

### Search strategy

We searched international primary research databases (PubMed, EMBASE and CENTRAL) from inception to 14 September 2021 and reference lists of relevant articles to identify all eligible studies. The following search strategy was used for all 3 databases:

1. ‘Pulmonary artery pulsatility index’ OR ‘PAPI’.
2. ‘Heart failure’ OR ‘ventricular failure’ OR ‘right ventricular failure’ OR ‘right heart failure’ OR ‘left ventricular failure’ OR ‘ventricular assist device’ OR ‘VAD’ OR ‘LVAD’ OR ‘Heartmate’ OR ‘Heartware’.
3. 1 AND 2.

An updated search was performed on 18 August 2022 using the same search strategy.

### Study selection and data extraction

Abstracts and then full-text articles of all identified were screened independently by 2 reviewers (Ivan H.W. Yim and Ayisha M. Khan-Kheil). All studies in patients with a durable LVAD, which contained PAPi data analysed against RHF or death, were included. RHF was author defined, including the Interagency Registry of Mechanically Assisted Circulatory Support (INTERMACS) definition of RHF [10]. Data were extracted independently by 2 reviewers (Ivan H.W. Yim and Ayisha M. Khan-Kheil) from the full-text publication and any disagreements were resolved by consensus. A full list of the data items and descriptors is available in the Supplementary Material. For all studies included, the Newcastle–Ottawa Scale [11] for assessing the quality of non-randomized studies was employed and based on the number of stars each study gained in each domain; this was then converted to the Agency for Healthcare Research and Quality standards of good, fair and poor.

### Statistical analysis

Statistical analysis was performed using IBM SPSS Statistics, Version 27.0 (Armonk, NY). All continuous data are expressed as medians with interquartile ranges and all categorical data are expressed as counts and percentages where applicable.

## RESULTS

**Figure 1:**
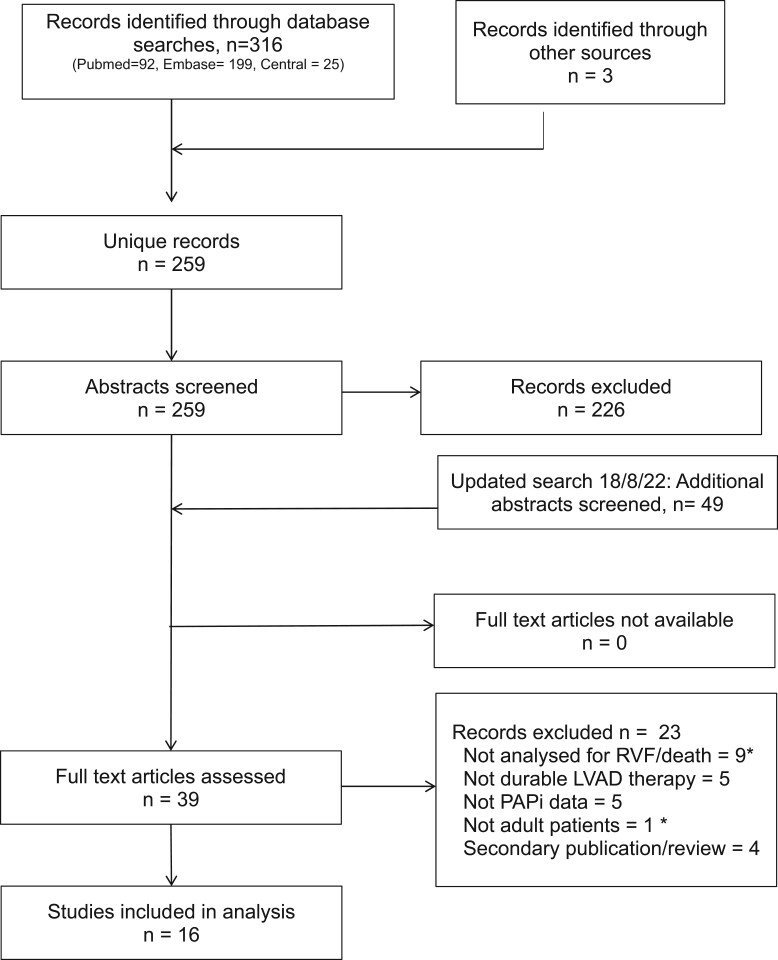
PRISMA flow diagram of study selection. *Multiple counting. LVAD: left ventricular assist device;; RHF: right heart failure.

The original search produced 259 unique records and we identified 14 studies reporting haemodynamic assessment in 20 634 adult patients with an implanted durable LVAD (Fig. 1). The updated search performed in August 2022 produced another 49 unique records and all full-text articles were sourced online or via national libraries; a total of 16 studies were included for analysis. Characteristics of the included studies are shown in Table 1. All studies were retrospective cohort studies originating from 4 countries, with 9 (56%) from the USA, 3 from Italy and 1 each from Japan, Germany, the Netherlands and Turkey. Articles were published in specialist heart failure, cardiothoracic surgery, transplantation or anaesthetic journals, and all were published in English.

**Table 1:** Summary of the 16 studies included in the full-text review

Authors	*N*	LVAD	PAPi associated with RHF?	PAPi cut-off for RHF or death	Summary
Grandin *et al.* [12]	151	Not reported	No	Not reported	Single-centre study. Low PAC combined with a high CVP: PCWP ratio was the strongest predictor for death at 6 months (HR 8.68, *P* < 0.001) and RHF (OR 4.74, *P* = 0.02). PAPI was not significantly associated with RHF (*P* = 0.10)
Morine *et al.* [13]	132	HM2, HVAD	Yes	<1.85 (RHF)	A lower PAPi was significantly associated with RHF *P* < 0.01. A PAPi < 1.85 provided 94% sensitivity and 81% specificity for predicting RHF and was superior to RAP: PCWP ratio, RVSWI and RAP alone.
Kang *et al.* [14]	83	HM2, HVAD	Yes	<2 (RHF)	PAPi was an independent predictor of RHF and RVAD implantation following LVAD therapy. A higher PAPi was associated with reduced risk for RVAD placement (OR 0.31, *P* < 0.0001). PAPi was more predictive of RVAD placement if inotropes were present at the time of catheterization (OR 0.21 vs 0.49). ROC analysis showed optimal sensitivity and specificity achieved using a PAPi threshold of 2.
Nitta *et al.* [15]	70	Nipro-VAD	Yes	<0.88 (RHF)	This study aimed to devise a scoring system for predicting RVAD placement following implantation of the Nipro-VAD paracoporeal device. Patients who required RVAD implantation postoperatively had a significantly lower PAPi (*P* = 0.001). The authors proposed a combination score using PVR > 4.5WU and RAP: PCWP > 0.8 as a scoring system for predicting RVAD requirement following paracoporeal LVAD therapy.
Loforte *et al.* [16]	258	HM2, HM3, HVAD, Jarvik 2000, Berlin Heart	Yes	<2 (RHF)	This study aimed to devise the ALMA risk score for predicting RHF following LVAD implantation. Within the haemodynamic data of this study, a PAPi of <2 was found to be associated with unplanned RVAD support (*P* = 0.001) and on multivariable logistic regression analysis PAPi < 2 had an OR of 3.3 (CI 1.7–6.1, *P* = 0.001). The ALMA score employs the following 5 variables: destination therapy intention, PAPI < 2, RVSWi <300 mmHg/ml/m^2^, RV:LV ratio > 0.75 and MELD-XI score >17. The authors proposed a score of 0–1 implies low risk for RVAD requirement and a score above 4 implies very high risk for requiring RVAD following LVAD implantation.
Raymer *et al.* [17]	216	HM2, HVAD	Yes	Not reported	This study reported the combination of TAPSE and HeartMate risk score (HMRS) as a scoring system to predict RHF following LVAD implantation. The RHF group had a lower PAPi (*P* = 0.001). ROC analysis showed PAPi had an AUC of 0.63 (*P* < 0.001). When the haemodynamic parameters were analysed combination of TAPSE with the HMRS was the best for predicting RHF compared to HMRS + PAPi and HMRS + sRVCPI.
Gudejko *et al.* [18]	85	HM2, HVAD	Yes	Not reported	The data used in this study were intraoperative haemodynamic parameters and also echocardiographic data. Higher CVP, lower pre-CPB and post-chest closure PAPi, post-CPB larger right atrial diameter, larger RVES area, lower FAC and lower TAPSE were all associated with severe RHF.
Muslem *et al.* [19]	375	HM2, HM3, HVAD	Yes	Not Reported	PAE was found to be the most robust haemodynamic parameter to predict RHF. PAPi was significantly lower in the Severe RHF group compared to no RHF (1.8 vs 2.2, *P* = 0.017).
Alfirevic *et al.* [20]	86	HM2, HM3, HVAD	No	Not reported	Intraoperative TAPSE measurement was the variable of interest in this study. As a secondary comparison, PAPi and the Michigan risk score were not significantly associated with severe RHF. Intraoperative TAPSE, Michigan risk score and PAPi were poor discriminators of RHF following LVAD therapy.
Sert *et al.* [21]	71	HM2, HM3, HVAD	No	Not reported	This study compared TAPSE, CVP: PCWP ratio, RVSWI, PAPi, Pennsylvania Score, Michigan score, CRITT score, ALMA score and the EUROMACS score. PAPi was not significantly lower in the group with postop RHF (*P* = 0.304). They concluded that only the EUROMACS and CRITT score had an ROC AUC above 0.7 and that the combination of TAPSE and the Pennsylvania score was found to be the most sensitive (85%) whereas TAPSE + Michigan score + CVP:PCWP ratio was the most specific (97%).
Benjamin *et al.* [22]	104	HM2, HVAD	No	Not reported	Primary end point was duration of inotropic support and the association with RVF. They found patients who were on long-term milrinone had a significantly increased risk of developing RHF post-LVAD insertion. PAPi was not significantly different between the RHF and the group without RVF post-LVAD implant (4 ± 3.9 vs 3.2 ± 2.3, *P* = 0.255)
Ruiz-Cano *et al.* [23]	80	HM3, HVAD	No	Not reported	PAPi was not significantly different between the early RHF group versus no early RHF (2.5 vs 3, *P* = 0.283). This study found that blood urea nitrogen >44.5 mg/dl and CVP/PCWP > 0.55 were the parameters with the strongest association with early RHF.
Guglin and Omar [24]	18608	Not reported	No data	Not reported	This was a retrospective cohort study looking at data from the INTERMACS database and primarily assessing RAP and its ability to predict death. RAP was the main predictor of mortality in LVAD recipients. PAPi was lower in non-survivors (*P* < 0.001), but RAP had superior discriminatory value with a difference in AUC of 0.0105 (*P* = 0.0052).
Gonzalez *et al.* [25]	315	HM2, HVAD	Yes	Optimal PAPi <3.3 (RHF); Delta PAPi <2.08 (Death at 6 months)	This study assessed the change in PAPi during preoperative haemodynamic optimization prior to LVAD implantation. The mean optimal PAPi was lower (*P* < 0.001) in the group that developed early RHF. A delta PAPI of <2.08 during optimization was associated with higher mortality at 180 days (*P* = 0.003).
Cacioli *et al.* [26]	75	HM2, HM3	Yes	Not reported	A lower PAPi was strongly associated with RHF following LVAD implant. This study also demonstrated in those who did not develop RHF post-LVAD had a significantly higher PAPi following vasodilator challenge at preop RHC (5.3 ± 3.9 vs 2.7 ± 1.3, *P* = 0.003). Furthermore, PAPi when combined with established risk scores provided incremental risk stratification for post-LVAD RHF.
Stricagnoli *et al.* [27]	38	HM3, Jarvik 2000	Yes	Not reported	PAPi was the most robust haemodynamic parameter which predicted post LVAD RHF (1.52 ± 0.26 vs 3.95 ± 3.39, *P* = 0.003) with an ROC AUC of 0.85.

ALMA: Antonio Loforte and Motalto Andrea; CVP: central venous pressure; CRITT: central venous pressure>15, severe right ventricular dysfunction, intubation, tricuspid regurgitation, tachycardia; EUROMACS: European Registry for Patients with Mechanical Circulatory Support; FAC: fractional area change; HR: heart rate; HVAD: HeartWare; INTERMACS: Interagency Registry of Mechanically Assisted Circulatory Support; LVAD: left ventricular assist device; MELD: Model for End-stage Liver Disease; PAC: pulmonary arterial compliance; PAPi: Pulmonary artery pulsatility index; PCWP: pulmonary capillary wedge pressure; RAP: right atrial pressure; RHF: right heart failure; ROC: receiver operating characteristic; RVAD: right ventricular assist device; RVES: right ventricular end systolic; RVSWI: right ventricular stroke work index; sRVCPI: simplified right ventricular contraction pressure index; TAPSE: tricuspid annular plane systolic excursion.

The study periods ranged from 2004 to 2021. The median number of patients in each study was 95 (interquartile range 78.8–226.5). The types of LVAD implanted were primarily intracorporeal continuous-flow devices, HeartMate (Abbott Laboratories, USA) II or III and HeartWare (Medtronic, USA), with 1 study evaluating the NIPRO-VAD (Nipro, Osaka, Japan) extracorporeal pulsatile pump [28]. Only 6 out of the 16 studies stated the goal of LVAD therapy (bridge to transplantation/candidacy or destination therapy).

Of the 16 studies, the primary outcome was (i) RHF or the event of right ventricular assist device implantation (*n* = 15) and (ii) mortality associated with right atrial pressure (RAP) (*n* = 1). All 16 studies reported on the timing of PAPI measurement in relation to LVAD implant. Fourteen studies performed right heart catheterization prior to LVAD implantation to record routine haemodynamic parameters including PAPI (although only 6 studies gave the exact timing in hours, days or months of RHC from LVAD implantation) and 2 studies measured PAPI intraoperatively at the time of LVAD implantation. Twelve studies did not specify medical therapy at the time of haemodynamic assessment (e.g. use of inotropes or intra-aortic balloon pump).

Guglin and Omar [24] analysed the INTERMACS database including 18 733 patients and found that RAP was a haemodynamic predictor of death with an receiver operating characteristic (ROC) curve area under the curve (AUC) of 0.55 (CI 0.539–0.562, *P* < 0.0001) and an RAP of 13 mmHg or higher had the highest combined sensitivity and specificity in predicting mortality. PAPI and other haemodynamic parameters were also analysed and compared against RAP. Survivors were found to have a significantly higher PAPI (3 ± 3.1 vs 2.6 ± 2.7; *P* < 0.001) but when compared to RAP, it was found to have a lower ROC curve AUC with a difference in areas of 0.0105, *P* = 0.005 and therefore RAP was found to be superior at predicting death.

Gonzalez *et al.* [25] studied PAPI at serial time points before LVAD implantation and during the period of medical optimization, hypothesizing that the magnitude of change of PAPI and other invasive haemodynamic measurements would provide an incremental risk stratification for RHF following LVAD therapy with a secondary end-point of death at 180 days. After optimizing their patients with a combination of diuretic, intravenous sodium nitroprusside, inotropes and non-durable mechanical circulatory support where appropriate, they found that an optimized preoperative PAPi of >3.33 was associated with a significant reduction in early RHF (*P* < 0.001). In patients with a change in PAPi (delta PAPi) of >2.08 during the optimization period (time period not specified), there was a significant reduction in 6-month mortality following LVAD implantation presumably from reduced early RHF. In a recently published study, Cacioli *et al.* [26] showed that PAPi following vasodilator challenge with sodium nitroprusside provided incremental risk stratification when combined with established risk scores (EUROMACS-RHF and CRITT). In this study, PAPi alone was significantly lower in patients who developed RHF post-LVAD implantation compared with those who did not have RVF (2.2 ± 1.3 vs 3.3 ± 1.5, *P* = 0.008). This was even more striking following vasodilator therapy (5.3 ± 3.9 vs 2.7 ± 3, *P* = 0.003) suggesting a higher PAPi following vasodilator therapy indicates more right ventricular reserve. Similarly to the study performed by Gonzalez *et al.*, this study reported a post-vasodilator challenge PAPi cut-off of 3.2 (with a sensitivity of 66% and a specificity of 68%) for right ventricular failure. Furthermore, the authors combined RV fractional area change and systolic pulmonary artery pressure with post-vasodilator challenge PAPi and found that it had an AUC of 0.949.

**Figure 2:**
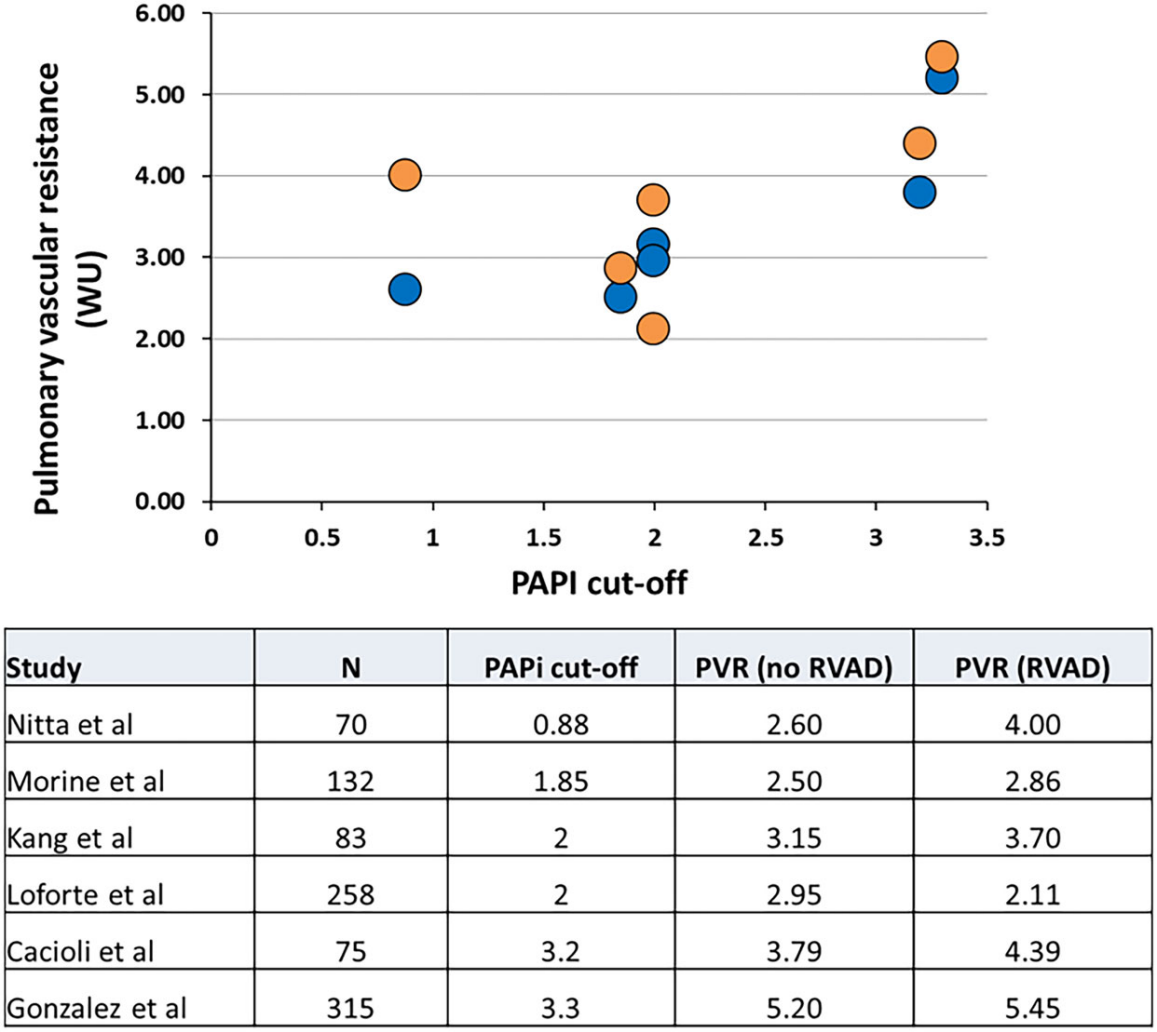
The relationship between PAPi cut-offs and PVR in the studies that reported on PAPi cut-offs. There is a direct correlation between PAPi and PVR (*r* = 0.6613, *P* = 0.019). PAPi: pulmonary artery pulsatility index; PVR: pulmonary vascular resistance.

The majority of studies used the INTERMACS definition to describe the end-point of RHF (Table 2). Fifteen studies reported RHF data relating to PAPi: in 10 [13–19, 25–27] of these studies, a lower PAPi was associated with RHF, but there was no significant association between PAPi and RHF in the other 5 studies [12, 20–23]. Six studies performed ROC analyses to determine the optimal PAPi cut-off for RHF following durable LVAD implantation, ranging from 0.88 to 3.3, with a mean of 2.2 and the ROC AUC values ranged from 0.70 to 0.94 with a mean of 0.80. All 6 studies provided data on PVR in patients with and without severe RHF. There was a direct relationship between the PAPi cut-offs and PVR for patients with/without severe RHF (*r* = 0.6613, *P* = 0.019) (Fig. 2).

**Table 2:** Definition of right heart failure used in the studies

Authors	RHF definition
Kang *et al.* [14]	INTERMACS definition
Nitta *et al.* [15]	RVAD implantation within 2 weeks of LVAD implantation
Raymer *et al.* [17]	Post-implant inotropic support >14 days, RVAD implantation or death within 14 days due to RHF
Loforte *et al.* [16]	Short or long term right sided MCS despite maximal dosage of continuous inotropic support and NO ventilation within 30 days of LVAD implantation.
Gonzalez *et al.* [25]	INTERMACS definition
Gudejko *et al.* [18]	INTERMACS definition
Alfirevic *et al.* [20]	INTERMACS definition
Morine *et al.* [13]	INTERMACS definition
Sert *et al.* [21]	INTERMACS definition
Grandin *et al.* [12]	RVAD or inotropic support >14 days or death from RHF within 14 days of LVAD implantation
Benjamin *et al.* [22]	RVAD or inotropic support >14 days within 30 days of LVAD implantation.
Muslem *et al.* [19]	INTERMACS definition
Ruiz-Cano *et al.* [23]	INTERMACS definition
Cacioli *et al.* [26]	INTERMACS definition
Stricagnoli *et al.* [27]	INTERMACS definition

INTERMACS definition of RHF: elevated central venous pressure >18 mmHg in the absence of elevated pulmonary capillary wedge pressure; RVAD implantation; or requirement of prolonged nitric oxide or inotropic therapy [39].

INTERMACS: Interagency Registry of Mechanically Assisted Circulatory Support; LVAD: left ventricular assist device; RHF: right heart failure; NO: nitric oxide; RVAD: right ventricular assist device.

In assessing the quality of the included studies using the Newcastle–Ottawa Scale, all achieved an Agency for Healthcare Research and Quality grading of good, with at least 1 star in each domain.

## DISCUSSION

PAPi has been evaluated as a parameter to predict the risk of RHF and death following LVAD implantation. Notable findings from this systematic review were: (i) lower PAPi measurements before LVAD implantation were associated with higher risk of RHF and/or death; (ii) the reported PAPi cut-offs for discriminating patients at risk of RHF and/or death varied by more than three-fold from 0.80 to 3.3; and (iii) the reported PAPi cut-offs were directly related to PVR.

PAPi is the ratio of PP over RAP (equation 1: PAPI = PP/RAP). Analogous to the charging of capacitors, a significant proportion of the right ventricular SV ‘charges’ the reservoir volume and increases pressure in the compliant pulmonary arteries in systole, which discharges during diastole. Pulmonary arterial compliance defines this relationship between an increase in blood volume (Δ*V*) and an increase in pressure in the pulmonary arterial system. In practice, PAC is difficult to measure because direct measurement of Δ*V* is not possible due to the continuous outflow from the arterial system. Therefore, in clinical practice, the ratio of SV/PP is used to determine PAC, accepting that this equation would overestimate the true PAC. Rearranging this equation, it can be appreciated that PAC and SV are the main determinants of PP (equation 2: PP = SV/PAC).

Pulmonary arterial compliance is determined by the prevailing distending pressure [i.e. mean pulmonary artery pressure (MPAP)] and by the elastic properties of the pulmonary arterial wall. The latter is mainly determined by the composition of elastin and collagen in the wall. Pulmonary arterial compliance decreases when MPAP increases in non-linear relationship [8] due to the nature of the stress–strain relationship; and MPAP itself is a function of PVR and left atrial pressure [or pulmonary artery wedge pressure (PAWP)], PVR, heart rate (HR) and SV {equation 3: MPAP = [PVR × (HR × SV)] + PAWP}.

**Figure 3:**
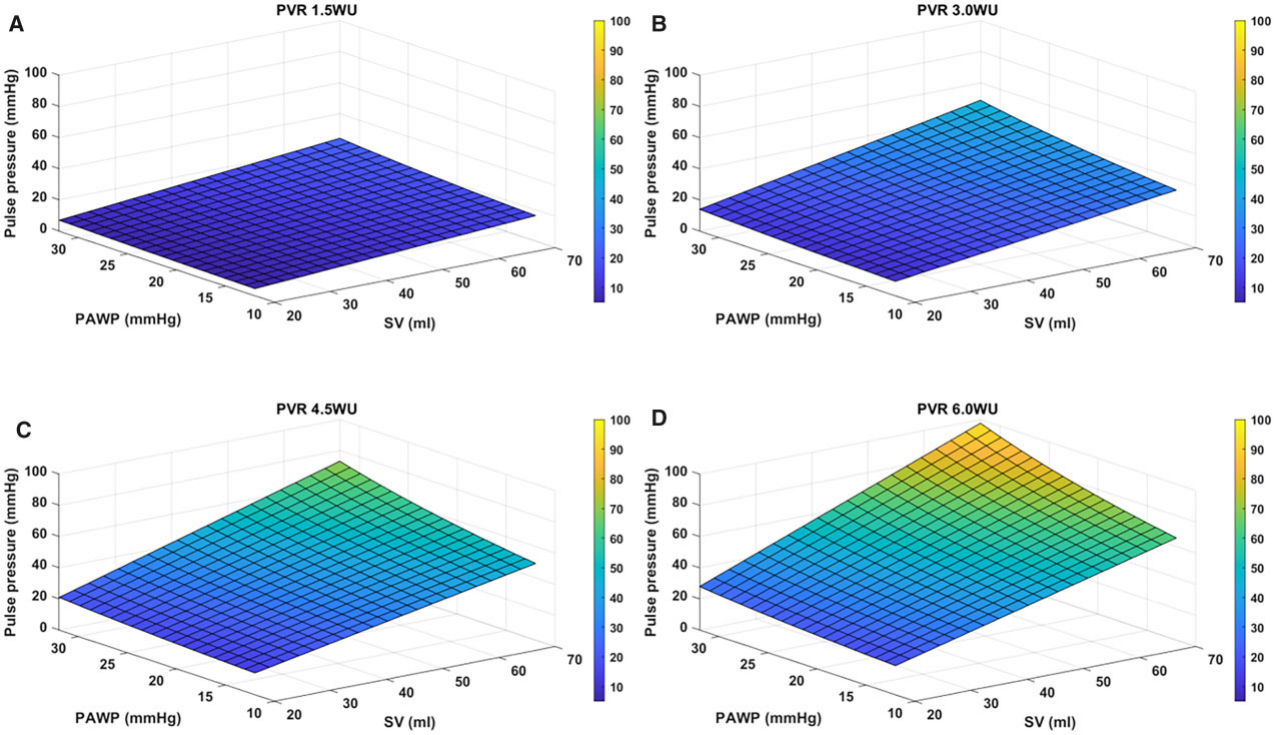
Pulmonary artery pulse pressure modelled over a range of pulmonary arterial wedge pressures, stroke volumes and pulmonary vascular resistances. Graphs A–D were modelled on a PVR of 1.5WU, 3WU, 4.5WU and 6WU, respectively, indicating increasing pulse pressure with increasing PVR. PVR: pulmonary vascular resistance.

Thus, PAC decreases as a result of: (i) an increasing distending pressure (MPAP) from a combination of increasing PVR, HR and SV; (ii) intrinsic changes in pulmonary arterial wall stiffness (vascular remodelling); and (iii) increase in left atrial pressure (or PAWP). Indeed, the hyperbolic relationship between PAC and PVR [29] and inverse relationship with PAWP are well characterized [30]. By extension, the pulmonary artery PP would be similarly dependent on SV, PAWP and PVR (Fig. 3). This would explain the observed relationship between the PAPi cut-off and PVR levels. We were unable to evaluate the relationship between HR, SV and PAPi, as the data were not reported in the studies.

The implications of these physiological considerations are three-fold. First, in the face of increasing PVR, the right ventricle mal-adapts to maintain relatively low RAP and normal SV; the latter results in disproportionately elevated PP, and by extension an increase in PAPi. The increase in PAPi is a result of progressive adverse remodelling and should not be misconstrued as an improvement in right heart function. Second, a significant drop in PAPi would only occur when the right ventricle uncouples from the pulmonary arterial system, with resultant drop in SV and rise in RAP. Third, because of the higher PVR and lower PAC, the PAPi level would remain higher in the setting of RHF related to pulmonary vascular disease compared to RHF due to primary right ventricular cardiomyopathy with low PVR and high PAC. In this context, Essandoh *et al.*’s recent description of a mean weighted PAPi of 2.17 as a cut-off to predict RHF post-LVAD implantation should be interpreted and applied with caution, and extrapolation to patients with other pulmonary haemodynamic profiles should be resisted.

Pulmonary artery PP is inversely related to PAC, which is inextricably related to PVR. An increase in PVR increases pulmonary artery PP at any given SV. In this regard, PVR is a determinant of pulmonary artery PP and PAPi and cannot be avoided in interpreting PAPi. The interpretation of PAPi must take into consideration the underlying disease pathophysiology.

In primary right ventricular dysfunction such as right ventricular infarction with low PVR, the compliant pulmonary arterial system would result in low pulmonary artery PP for a given SV and RAP. In contrast, PAPi must be higher at the same SV and RAP in high conditions, such as primary arterial hypertension.

As an illustration, we describe 2 clinical scenarios:

Patient A, assuming a RAP of 10mmHg, has a normal PVR and normal PAC of 5ml/mmHg. Even with supranormal stroke volume of 100ml (pulse pressure=20mmHg), his PAPi would only be 2. Conversely, patient B has a high RAP of 20mmHg and a low PAC of 1ml/mmHg due to pulmonary vascular disease. Even with a RAP that is two times higher and half the stroke volume (50ml, pulse pressure=50mmHg), patient B has a higher PAPi of 2.5. Despite the lower PAPi, it is physiologically implausible to suggest that patient A has more severe right heart failure compared to patient B.

These 2 clinical scenarios highlight the key message in our systematic review—the same PAPi cut-off cannot be applied in different clinical conditions, and a single cut-off averaged from a number of heterogenous studies is likely to be misleading. Interpretation of PAPi requires an appreciation of the underlying disease and pathophysiology. We have developed a calculator available in the Supplementary Material, which shows the mechanisms for the observed PAPi depending on the SV and PVR.

It should be noted that other factors may also contribute to the variable cut-offs and discriminatory value in patients undergoing LVAD implantation. First, there are multiple causes of RHF following LVAD implantation, including technical/surgical factors. Second, the definition of severe RHF is related to the postoperative management strategy. The clinical threshold for the use of right ventricular assist device may explain the variable incidence of severe RHF reported in the literature. Thirdly, the timing of assessment relative to LVAD implantation was not uniformly described in the studies. PAPi would change depending on the effects of medical therapy, including diuretics, vasodilators and inotropes, as reported by Gonzalez *et al.* [25]. The assessment of PAPi closer to the time of LVAD implantation may have greater discriminatory value.

### Limitations

With only 2 publications reporting on death in relation to PAPi this precludes meta-analysis and hence the descriptive nature of this systematic review. However, we believe that our description of the relationship between PAPi and PVR (and therefore a single PAPi cut-off value cannot be defined for heterogenous conditions) is not widely appreciated, yet highly relevant to clinicians, especially with greater adoption of PAPi into clinical practice.

## CONCLUSION

In conclusion, lower PAPi is associated with a higher risk of RHF and mortality in patients with durable LVAD therapy. However, PAPi is inherently related to PVR and the different PVR levels may have resulted in the variable PAPi cut-offs described in the studies. The PVR level should be taken into consideration in the interpretation of PAPi.

## Supplementary Material

ivad068_Supplementary_Data

## Data Availability

All relevant data are within the article and its supporting information files.

## ABBREVIATIONS

HR: Heart rate
INTERMACS: Interagency Registry of Mechanically Assisted Circulatory Support
LVAD: Left ventricular assist device
MPAP: Mean pulmonary arterial pressure
PAC: Pulmonary artery compliance
PAPi: Pulmonary artery pulsatility index
PAWP: Pulmonary artery wedge pressure
PP: Pulse pressure
PVR: Pulmonary vascular resistance
RAP: Right atrial pressure
RHF: Right heart failure
SV: Stroke volume
